# No detrimental effect of a positive family history on postoperative upgrading and upstaging in men with low risk and favourable intermediate-risk prostate cancer: implications for active surveillance

**DOI:** 10.1007/s00345-020-03485-5

**Published:** 2020-10-13

**Authors:** Kathleen Herkommer, Nikola Maier, Donna P. Ankerst, Stefan Schiele, Jürgen E. Gschwend, Valentin H. Meissner

**Affiliations:** 1 Department of Urology, School of Medicine, Klinikum Rechts der Isar, Technical University of Munich, Ismaninger Strasse 22, 81675 Munich, Germany; 2 Department of Mathematics and Life Sciences, Technical University of Munich, Munich, Germany

**Keywords:** Active surveillance, Family history, Fatal family history, Favourable intermediate-risk prostate cancer, Upgrading, Upstaging

## Abstract

**Purpose:**

To assess whether a first-degree family history or a fatal family history of prostate cancer (PCa) are associated with postoperative upgrading and upstaging among men with low risk and favourable intermediate-risk (FIR) PCa and to provide guidance on clinical decision making for active surveillance (AS) in this patient population.

**Methods:**

Participants in the German Familial Prostate Cancer database diagnosed from 1994 to 2019 with (1) low risk (clinical T1c–T2a, biopsy Gleason Grade Group (GGG) 1, PSA < 10 ng/ml), (2) Gleason 6 FIR (clinical T1c–T2a, GGG 1, PSA 10–20 ng/ml), and (3) Gleason 3 + 4 FIR (clinical T1c–T2a, GGG 2, PSA < 10 ng/ml) PCa who were subsequently treated with radical prostatectomy (RP) were analysed for upgrading, defined as postoperative GGG 3 tumour or upstaging, defined as pT3–pT4 or pN1 disease at RP. Logistic regression analysis was used to assess whether PCa family history was associated with postoperative upgrading or upstaging.

**Results:**

Among 4091 men who underwent RP, mean age at surgery was 64.4 (SD 6.7) years, 24.7% reported a family history, and 3.4% a fatal family history. Neither family history nor fatal family history were associated with upgrading or upstaging at low risk, Gleason 6 FIR, and Gleason 3 + 4 FIR PCa patients.

**Conclusion:**

Results from the current study indicated no detrimental effect of family history on postoperative upgrading or upstaging. Therefore, a positive family history or fatal family history of PCa in FIR PCa patients should not be a reason to refrain from AS in men otherwise suitable.

**Electronic supplementary material:**

The online version of this article (10.1007/s00345-020-03485-5) contains supplementary material, which is available to authorized users.

## Introduction

Active surveillance (AS) has emerged as a standard initial management option for low-risk prostate cancer (PCa) to reduce overtreatment and treatment-associated morbidity. Recently, National Comprehensive Cancer Network guidelines recommended AS as an option for men with favourable intermediate-risk (FIR) PCa [1]. However, whether AS can be safely extended to FIR PCa patients remains a matter of debate [2–4]. Risk stratifications prior to treatment decisions are primarily based on biopsy Gleason Grade Group (GGG), clinical stage, and prostate-specific antigen (PSA) levels. Significant sampling error and morbidity associated with prostate biopsy complicates differentiation between aggressive and indolent disease so that post-radical prostatectomy (RP) upgrading and upstaging is common [5–7]. Recently, multi-parametric magnetic resonance imaging (mpMRI) showed improvements in risk stratification of men on AS and was recommended for enhancing enrolment and monitoring decision [8–10].

A series of prior studies have suggested older age and African American race to be associated with higher rates of postoperative upgrading and upstaging [6, 11, 12]. Whether a first-degree family history of PCa correlates with higher rates of upgrading and upstaging has been less well-studied, with only one study reporting no association among low-risk familial PCa patients [13]. Whether men with FIR PCa and family history or fatal family history are at particularly high risk of harbouring undetected high-grade or high-stage disease has to date not been investigated. Patients with a familial burden of lethal or advanced PCa might experience higher levels of anxiety and may, therefore feel uncomfortable with AS.

The aim of this study was to assess whether family history or fatal family history is associated with rates of postoperative upgrading and upstaging among men with low risk and FIR PCa to provide guidance on clinical decision making for AS for these patients.

## Patients and methods

### Database and patient population

The prospective multicentre German Familial Prostate Cancer study has been recruiting and surveying newly diagnosed PCa patients independent of their family history since 1994 [14, 15]. Briefly, patients are referred by attending urologists and cooperating clinics throughout Germany. Patients report sociodemographic data whereas clinicopathological data are verified by a histopathological report or a doctor’s letter. Informed consent is obtained from each patient. The study was approved by the ethical review committee of the Technical University of Munich.

Patients from the study diagnosed between 1994 and 2019 with low risk or FIR PCa and treated with RP were identified. Patients with neoadjuvant or other first-line therapies were excluded. Eligible patients were classified by AUA risk strata [16]. Low-risk PCa was defined as clinical T1c–T2a, biopsy GGG 1, and PSA < 10 ng/ml. FIR PCa was further subclassified into Gleason 6 FIR PCa (clinical T1c–T2a, GGG 1, PSA 10–20 ng/ml) and Gleason 3 + 4 FIR PCa (clinical T1c–T2a, GGG 2, PSA < 10 ng/ml). Gleason score was assigned according to the 2005 International Society of Urological Pathology Consensus Conference on Gleason Grading of Prostatic Carcinoma [17]. Accordingly, newer GGG designations were applied.

Sociodemographic and clinical data included age at surgery, PSA at diagnosis, digital rectal examination (DRE), adjuvant radiotherapy, and adjuvant hormone therapy. Cancer family history included family history of PCa and other cancer, fatal family history (defined as at least one first-degree relative who died of PCa), and secondary cancer of the patient. family history of PCa was stratified into hereditary PCa according to the Johns Hopkins criteria [18], first-degree relatives with PCa, and non-familial first-degree PCa. Pathological data included pathological tumour stage, pathological node stage, surgical margin, and pathological GGG. Data on patients diagnosed before 2002 were adjusted to the UICC TNM classification 2002.

### Statistical analysis

Participant characteristics were presented by descriptive statistics. Primary outcomes were upgrading, defined as postoperative GGG 3 tumour in RP and upstaging, defined as pT3–pT4 or pN1 disease at RP. Single logistic regression analysis was employed to determine univariate associations of family history, fatal family history, and all patient risk factors with outcomes. Multiple logistic regression with backward elimination (selection level 5%) was subsequently implemented to identify independent effects from other risk factors. Odds ratios (OR) with 95% confidence intervals (CI) and two-sided *p* values were reported, with statistical significance set at the 0.05 level. All analyses were conducted using SAS 9.4.

## Results

### Study population

Table 1 depicts sociodemographic, clinical, family history, and pathological characteristics of the 4091 patients eligible for analysis. Mean age at surgery was 64.1 (SD = 6.6) years and mean PSA at diagnosis was 7.0 (SD = 3.3) ng/ml. 25.3% reported a first-degree family history of PCa and 3.8% had a fatal family history of PCa. Overall, 63.7%, 15.4%, and 20.9% of patients had low risk, Gleason 6 FIR, and Gleason 3 + 4 FIR PCa, respectively. Of low-risk PCa patients, 7.2% and 12.0% were postoperatively upgraded and upstaged, respectively, and these numbers increased to 10.6% and 24.1% for Gleason 6 FIR PCa patients and 13.8% and 16.6% for Gleason 3 + 4 FIR PCa patients.

**Table 1:** Patient characteristics (*n* = 4091)

Preoperative risk stratification, *n* (%)	
Low risk PCa	2607 (63.7)
Gleason 6 FIR PCa	630 (15.4)
Gleason 3 + 4 FIR PCa	854 (20.9)
Upgrading* in low risk PCa patients, *n* (%)	
Yes	183 (7.2)
No	2356 (92.8)
Upgrading* in Gleason 6 FIR PCa patients, *n* (%)	
Yes	67 (10.6)
No	563 (89.4)
Upgrading* in Gleason 3 + 4 FIR PCa patients, *n* (%)	
Yes	118 (13.8)
No	736 (86.2)
Upstaging** in low risk PCa patients, *n* (%)	
Yes	313 (12.0)
No	2294 (88.0)
Upstaging** in Gleason 6 FIR PCa patients, *n* (%)	
Yes	152 (24.1)
No	478 (75.9)
Upstaging** in Gleason 3 + 4 FIR PCa patients, *n* (%)	
Yes	142 (16.6)
No	712 (83.4)
Age at surgery, mean (SD), years	64.1 (6.6)
≤ 55, *n* (%)	389 (9.5)
> 55 to ≤ 65, *n* (%)	1741 (42.6)
> 65, *n* (%)	1961 (47.9)
Family history of PCa, *n* (%)	
Non	3934 (74.7)
First degree	748 (18.3)
Hereditary	286 (7.0)
Fatal family history of PCa, *n* (%)	
Non	3934 (96.2)
Yes	157 (3.8)
Other cancer family history, *n* (%)	
Non	2063 (50.4)
Yes	2027 (49.6)
Secondary cancer, *n* (%)	
Non	3565 (87.1)
Urologic cancer	139 (3.4)
Non-urologic cancer	387 (9.5)
PSA at diagnosis, mean (SD), ng/mL	7.0 (3.3)
≤ 4, *n* (%)	475 (11.6)
> 4 to ≤ 10, *n* (%)	3010 (73.6)
> 10 to ≤ 20, *n* (%)	606 (14.8)
DRE, *n* (%)	
Non-suspicious	3218 (78.7)
Suspicious	873 (21.3)
Pathological tumour stage, *n* (%)	
≤ pT2c	3509 (85.8)
pT3a	437 (10.7)
pT3b	124 (3.0)
pT4	21 (0.5)
Pathological node stage, *n* (%)	
pN0	4025 (98.5)
pN1	61 (1.5)
Surgical margin, *n* (%)	
R0	2651 (88.7)
R1	339 (11.3)
Pathological Gleason Grade Group, *n* (%)	
1	2326 (58.6)
2/3	120 (3.0)
2	1205 (30.4)
3	220 (5.5)
4	97 (2.5)
Adjuvant radiotherapy, *n* (%)	
Yes	158 (3.9)
No	3933 (96.1)
Adjuvant hormone therapy, *n* (%)	
Yes	107 (2.6)
No	3984 (97.4)

*PCa* prostate cancer, *SD* standard deviation, *FIR* favourable intermediate risk, *PSA* prostate-specific antigen, *DRE* digital rectal examination

*Upgrading was defined as postoperative Gleason Grade Group 3 tumour in radical prostatectomy

**Upstaging was defined as pT3–pT4 or pN1 disease at radical prostatectomy

### Predictors of upgrading and upstaging in men with low-risk PCa

On single logistic regression analysis, family history of PCa and other cancer as well as fatal family history were not associated with both upgrading and upstaging in low-risk PCa patients (Table 2). In the multiple analysis, higher age (OR 1.06, 95% CI 1.03–1.08) and higher PSA at diagnosis (OR 1.14, 95% CI 1.05–1.23) were associated with postoperative upgrading. Secondary non-urologic cancers (OR 0.52, 95% CI 0.28–0.97) were associated with a lower risk of upgrading. Higher PSA at diagnosis (1.18, 95% CI 1.11–1.26) was the only factor associated with postoperative upstaging.

**Table 2:** Single and multiple logistic regression analysis of upgrading and upstaging in men with low risk PCa

Factors	Upgrading						Upstaging					
	Single regression			Multiple regression^a^			Single regression			Multiple regression^a^		
	OR	95% CI	*p* value	OR	95% CI	*p* value	OR	95% CI	*p* value	OR	95% CI	*p* value
Age at surgery			< 0.001			< 0.001			0.083			
Continuous	1.06	[1.03; 1.09]		1.06	[1.03; 1.08]		1.02	[1.00; 1.04]				
Family history of PCa (ref: non)			0.893						0.064			
First degree	0.92	[0.62; 1.38]					1.39	[1.04; 1.86]				
Hereditary	0.91	[0.52; 1.61]					1.27	[0.84; 1.93]				
Fatal family history of PCa (ref: non)			0.213						0.092			
Yes	0.53	[0.19; 1.45]					1.57	[0.93; 2.64]				
Other cancer family history (ref: non)			0.515						0.824			
Yes	1.11	[0.82; 1.49]					0.97	[0.77; 1.23]				
Secondary urologic cancer (ref: non)			0.225						0.486			
Urologic cancer	0.49	[0.15; 1.56]					1.25	[0.67; 2.33]				
Secondary non-urologic cancer (ref: non)			0.061			0.041			0.951			
Non-urologic cancer	0.55	[0.30; 1.03]		0.52	[0.28; 0.97]		1.01	[0.69; 1.50]				
PSA at diagnosis (ng/ml)			< 0.001			0.001			< 0.001			< 0.001
Continuous	1.15	[1.07; 1.25]		1.14	[1.05; 1.23]		1.18	[1.11; 1.26]		1.18	[1.11; 1.26]	
DRE (ref: non-suspicious)			0.559						0.175			
Suspicious	1.11	[0.78; 1.59]					0.81	[0.60; 1.10]				

*OR* odds ratio, *CI* confidence interval, *PCa* prostate cancer, *PSA* prostate-specific antigen, *DRE* digital rectal examination

^a^With backward elimination (selection level 5%)

### Predictors of upgrading and upstaging in men with FIR PCa

In single logistic regression analyses, neither family history nor fatal family history were associated with upgrading and upstaging in men with Gleason 6 FIR and Gleason 3 + 4 FIR PCa (Tables 3, 4). In multiple regression analyses, higher PSA level at diagnosis (1.14, 95% CI 1.03–1.26) and suspicious DRE (1.73, 95% CI 1.08–2.78) were associated with upgrading among Gleason 3 + 4 FIR PCa patients. Higher PSA level at diagnosis (1.16, 95% CI 1.05–1.27) was associated with an increased risk of upstaging in Gleason 3 + 4 FIR PCa patients (Table 4).

**Table 3:** Single and multiple logistic regression analysis of upgrading and upstaging in men with Gleason 6 favourable intermediate risk PCa

Factors	Upgrading						Upstaging					
	Single regression			Multiple regression^a^			Single regression			Multiple regression^a^		
	OR	95% CI	*p* value	OR	95% CI	*p* value	OR	95% CI	*p* value	OR	95% CI	*p* value
Age at surgery			0.550						0.936			
Continuous	1.01	[0.97; 1.06]					1.00	[0.97; 1.03]				
Family history of PCa (ref: non)			0.194						0.117			
First degree	0.45	[0.19; 1.07]					0.56	[0.32; 0.98]				
Hereditary	1.03	[0.35; 3.04]					1.09	[0.49; 2.41]				
Fatal family history of PCa (ref: non)			0.810						0.292			
Yes	0.83	[0.19; 3.66]					0.52	[0.15; 1.77]				
Other cancer family history (ref: non)			0.281						0.083			
Yes	1.33	[0.80; 2.21]					1.38	[0.96; 2.00]				
Secondary urologic cancer (ref: non)			0.769						0.473			
Urologic cancer	1.21	[0.35; 4.17]					1.39	[0.56; 3.46]				
Secondary non-urologic cancer			0.233						0.876			
Non-urologic cancer	1.63	[0.73; 3.64]					1.05	[0.55; 2.03]				
PSA at diagnosis (ng/ml)			0.283						0.943			
Continuous	1.05	[0.96; 1.16]					1.00	[0.94; 1.08]				
DRE (ref: non-suspicious)			0.733						0.238			
Suspicious	1.10	[0.63; 1.94]					0.77	[0.50; 1.19]				

*OR* odds ratio, *CI* confidence interval, *PCa* prostate cancer, *PSA* prostate-specific antigen, *DRE* digital rectal examination

^a^With backward elimination (selection level 5%)

**Table 4:** Single and multiple logistic regression analysis of upgrading and upstaging in men with Gleason 3 + 4 favourable intermediate risk PCa

Factors	Upgrading						Upstaging					
	Single regression			Multiple regression^a^			Single regression			Multiple regression^a^		
	OR	95% CI	*p* value	OR	95% CI	*p* value	OR	95% CI	*p* value	OR	95% CI	*p* value
Age at surgery			0.052						0.029			
Continuous	1.03	[1.00; 1.06]					1.03	[1.01; 1.06]				
Family history of PCa (ref: non)			0.450						0.221			
First degree	0.73	[0.44; 1.22]					1.04	[0.67; 1.61]				
Hereditary	0.78	[0.30; 2.02]					0.52	[0.18; 1.48]				
Fatal family history of PCa (ref: non)			0.233						0.525			
Yes	0.42	[0.10; 1.76]					0.71	[0.25; 2.05]				
Other cancer family history (ref: non)			0.729						0.953			
Yes	0.93	[0.63; 1.38]					1.01	[0.70; 1.45]				
Secondary urologic cancer (ref: non)			0.783						0.064			
Urologic cancer	1.15	[0.43; 3.04]					0.15	[0.02; 1.11]				
Secondary non-urologic cancer (ref: non)			0.698						0.948			
Non-urologic cancer	1.14	[0.58; 2.24]					1.02	[0.53; 1.95]				
PSA at diagnosis (ng/ml)			0.013			0.013			0.003			0.003
Continuous	1.14	[1.03; 1.27]		1.14	[1.03; 1.26]		1.16	[1.05; 1.27]		1.16	[1.05; 1.27]	
DRE (ref: non-suspicious)			0.023			0.022			0.192			
Suspicious	1.73	[1.08; 2.76]		1.73	[1.08; 2.78]		0.70	[0.41; 1.20]				

*OR* odds ratio, *CI* confidence interval, *PCa* prostate cancer, *PSA* prostate-specific antigen, *DRE* digital rectal examination

^a^With backward elimination (selection level 5%)

Additionally, a separate regression analysis for upgrading and upstaging of the entire cohort was conducted to gain more statistical power. In this analysis, neither a positive family history nor a fatal family history of PCa were associated with a higher likelihood of upgrading and upstaging, respectively (Supplementary Table 1). Furthermore, since upgrading from GGG 1 to GGG 2 is relevant concerning treatment decisions and patient counselling, we analysed additionally any upgrading from GGG 1 to GGG 2 in a separate regression analysis finding again no association between upgrading and a positive family history or a fatal family history in the multiple regression model (Supplementary Table 2).

## Discussion

AS has been increasingly accepted as a safe approach to low-risk PCa with long-term outcomes similar to those of men with curative treatment strategies in randomized controlled trials [19] and in prospectively maintained cohorts [20, 21]. However, a major limitation is that a significant proportion of patients harbour occult higher-grade and higher-stage disease, which might lead to anxiety and refusal of AS initiation in at risk groups such as in men with a familial burden of PCa.

Results of this study showed that family history, defined as one or more first-degree relatives diagnosed with PCa including those with hereditary PCa, and fatal family history were not associated with postoperative upgrading or upstaging in men with low risk and FIR PCa. Since FIR patients represent a heterogeneous group [22], they were divided into Gleason 6 FIR and Gleason 3 + 4 FIR PCa patients for more specific association analysis. However, no detrimental effect of family history was observed. Therefore, FIR PCa patients with family history could be reassured that their familial burden of PCa is not associated with a higher rate of occult high-grade or high-stage disease compared to men with no family history and that AS might be a reasonable treatment option. Clearly, men with FIR disease should be informed of the risks of harbouring undetected higher-grade and higher-stage disease, since rates of postoperative upgrading and upstaging were higher in both Gleason 6 and Gleason 3 + 4 FIR patients compared to low-risk patients (upgrading: 13.8% and 10.6% vs. 7.2%; *p* < 0.001; upstaging: 24.1% and 16.6% vs. 12.0%; *p* < 0.001), which could impact secondary therapies and increase risk of PCa progression. Reported rates of upgrading and upstaging are mostly consistent with results in the recent literature [12, 23–25]. Interestingly, a higher PSA level at diagnosis was a significant predictor of both upgrading and upstaging in low risk and Gleason 3 + 4 FIR PCa, but not in Gleason 6 FIR PCa. The reasons remain unclear; however, the results indicate that the FIR PCa group is a heterogeneous group which warrants further investigation.

In line with previous findings [26], this study found an association between a suspicious DRE and a higher risk of postoperative upgrading in Gleason 3 + 4 FIR PCa patients, which emphasises the important role of DRE despite recent controversy over the usefulness in PCa staging. Indeed, emerging evidence suggests that sensitivity, specificity, negative and positive predictive values of DRE to detect clinically significant PCa were better when PSA levels were lower [27]. This result is consistent with findings here since PSA levels of the Gleason 3 + 4 FIR group were lower compared to the Gleason 6 FIR group (6.1 ng/ml vs. 13.0 ng/ml; *p* < 0.001; data not shown).

In the present analysis, older age was only associated with a higher risk of upgrading among low-risk PCa patients. Contradictory results have been likewise reported in the literature [24, 26, 28, 29]. Nevertheless, this factor merits further investigation to avoid overtreatment in elderly patients who have a higher risk of death from competing causes when treated with definitive curative treatment [30].

The strength of this study is the large nationwide, population-based sample with verified, complete, and detailed information concerning family history and fatal family history of PCa. Limitations include first that mpMRI and targeted fusion biopsies were rarely used during the study period and hence were excluded from the analysis. Second, patients were selected for RP and hence may not be representative of all PCa patients as a result of selection bias. Third, information on prognostic factors of unfavourable outcomes, such as perineural invasion, percentage PCa in a core, and PSA density were lacking and not incorporated in the analysis. Furthermore, biopsies were neither performed according to a uniform protocol for biopsy core collection nor centrally reviewed. Additionally, biopsy and prostatectomy specimen were not consistently reviewed centrally by the same uropathologist, increasing the risk of inter-observer variation of grading. Finally, the study was retrospective and subject to the usual limitations of retrospective analyses.

## Conclusions

Results of the present study showed no detrimental effect of family history on postoperative upgrading or upstaging in men with low risk and FIR PCa. Patients with a fatal family history of PCa had likewise no increased risk of postoperative upgrading or upstaging. Separate evaluation in Gleason 6 FIR and Gleason 3 + 4 FIR PCa patients confirmed the conclusions. Family history or fatal family history in FIR PCa patients should not be a reason to refrain from AS that is otherwise suitable.

## Electronic supplementary material

Below is the link to the electronic supplementary material.

Supplementary file1 (DOCX 15 kb)

Supplementary file2 (DOCX 14 kb)

## References

[1] Mohler JL, Armstrong AJ, Bahnson RR (2016). Prostate cancer, Version 1.2016. J Natl Compr Canc Netw.

[2] Ploussard G, Isbarn H, Briganti A (2015). Can we expand active surveillance criteria to include biopsy Gleason 3+4 prostate cancer? A multi-institutional study of 2323 patients. Urol Oncol.

[3] Yamamoto T, Musunuru HB, Vesprini D (2016). Metastatic prostate cancer in men initially treated with active surveillance. J Urol.

[4] Raldow AC, Zhang D, Chen MH, Braccioforte MH, Moran BJ, D'Amico AV (2015). Risk Group and Death From Prostate Cancer: Implications for Active Surveillance in Men With Favorable Intermediate-Risk Prostate Cancer. JAMA Oncol.

[5] Corcoran NM, Hovens CM, Hong MK (2012). Underestimation of Gleason score at prostate biopsy reflects sampling error in lower volume tumours. BJU Int.

[6] Morlacco A, Cheville JC, Rangel LJ, Gearman DJ, Karnes RJ (2017). Adverse disease features in Gleason Score 3 + 4 "favorable intermediate-risk" prostate cancer: implications for active surveillance. Eur Urol.

[7] Kaye DR, Qi J, Morgan TM (2019). Pathological upgrading at radical prostatectomy for patients with Grade Group 1 prostate cancer: implications of confirmatory testing for patients considering active surveillance. BJU Int.

[8] Ploussard G, Beauval JB, Lesourd M (2020). Performance of systematic, MRI-targeted biopsies alone or in combination for the prediction of unfavourable disease in MRI-positive low-risk prostate cancer patients eligible for active surveillance. World J Urol.

[9] Mamawala MK, Meyer AR, Landis PK (2020). Utility of multiparametric magnetic resonance imaging in the risk stratification of men with Grade Group 1 prostate cancer on active surveillance. BJU Int.

[10] Björnebo L, Olsson H, Nordström T (2020). Predictors of adverse pathology on radical prostatectomy specimen in men initially enrolled in active surveillance for low-risk prostate cancer. World J Urol.

[11] Maurice MJ, Sundi D, Schaeffer EM, Abouassaly R (2017). Risk of pathological upgrading and up staging among men with low risk prostate cancer varies by race: results from the National Cancer Database. J Urol.

[12] Dinh KT, Mahal BA, Ziehr DR (2015). Incidence and predictors of upgrading and up staging among 10,000 contemporary patients with low risk prostate cancer. J Urol.

[13] Jansson F, Folkvaljon F, Stattin P, Bratt O, Akre O (2020). Risk of postoperative up staging or upgrading among men with low risk familial prostate cancer. J Urol.

[14] Meissner VH, Strüh JGH, Kron M (2020). The role of fatal family history and mode of inheritance in prostate cancer for long-term outcomes following radical prostatectomy. World J Urol.

[15] Paiss T, Herkommer K, Chab A (2002). Familial prostate carcinoma in Germany. Urologe A.

[16] Sanda MG, Cadeddu JA, Kirkby E (2018). Clinically localized prostate cancer: AUA/ASTRO/SUO Guideline. Part I: risk stratification, shared decision making, and care options. J Urol.

[17] Epstein JI, Zelefsky MJ, Sjoberg DD (2016). A contemporary prostate cancer grading system: a validated alternative to the Gleason Score. Eur Urol.

[18] Carter BS, Bova GS, Beaty TH (1993). Hereditary prostate cancer: epidemiologic and clinical features. J Urol.

[19] Hamdy FC, Donovan JL, Lane JA (2016). 10-year outcomes after monitoring, surgery, or radiotherapy for localized prostate cancer. N Engl J Med.

[20] Klotz L, Vesprini D, Sethukavalan P (2015). Long-term follow-up of a large active surveillance cohort of patients with prostate cancer. J Clin Oncol.

[21] Tosoian JJ, Mamawala M, Epstein JI (2015). Intermediate and longer-term outcomes from a prospective active-surveillance program for favorable-risk prostate cancer. J Clin Oncol.

[22] Beauval JB, Ploussard G, Cabarrou B (2017). Improved decision making in intermediate-risk prostate cancer: a multicenter study on pathologic and oncologic outcomes after radical prostatectomy. World J Urol.

[23] Patel HD, Gupta M, Tosoian JJ, Carter HB, Partin AW, Epstein JI (2018). Subtyping the risk of intermediate risk prostate cancer for active surveillance based on adverse pathology at radical prostatectomy. J Urol.

[24] Yang DD, Mahal BA, Muralidhar V (2019). Risk of upgrading and upstaging among 10000 patients with Gleason 3+4 favorable intermediate-risk prostate cancer. Eur Urol Focus.

[25] Gearman DJ, Morlacco A, Cheville JC, Rangel LJ, Karnes RJ (2018). Comparison of pathological and oncologic outcomes of favorable risk Gleason Score 3 + 4 and low risk Gleason Score 6 prostate cancer: considerations for active surveillance. J Urol.

[26] Vellekoop A, Loeb S, Folkvaljon Y, Stattin P (2014). Population based study of predictors of adverse pathology among candidates for active surveillance with Gleason 6 prostate cancer. J Urol.

[27] Herrera-Caceres JO, Wettstein MS, Goldberg H (2020). Utility of digital rectal examination in a population with prostate cancer treated with active surveillance. Can Urol Assoc J.

[28] De Nunzio C, Brassetti A, Simone G (2018). Metabolic syndrome increases the risk of upgrading and upstaging in patients with prostate cancer on biopsy: a radical prostatectomy multicenter cohort study. Prostate Cancer Prostatic Dis.

[29] Herlemann A, Buchner A, Kretschmer A, Apfelbeck M, Stief CG, Gratzke C, Tritschler S (2017). Postoperative upgrading of prostate cancer in men ≥ 75 years: a propensity score-matched analysis. World J Urol.

[30] Daskivich TJ, Chamie K, Kwan L (2011). Comorbidity and competing risks for mortality in men with prostate cancer. Cancer.

