# Supplementary material for: No detrimental effect of a positive family history on postoperative upgrading and upstaging in men with low risk and favourable intermediate-risk prostate cancer: implications for active surveillance
Source: World J Urol. 2020 Oct 13;39(7):2499–506. doi: 10.1007/s00345-020-03485-5 (PMC8332649; doi:10.1007/s00345-020-03485-5)
Supplement: Supplementary file 1 — Supplementary file1 (DOCX 15 kb) [file 345_2020_3485_MOESM1_ESM.docx]

| **Supplementary Table 1** Single and multiple regression analysis of upgrading and upstaging in the entire cohort (n = 4,091) | | | | | | | | | | | | | |
| --- | --- | --- | --- | --- | --- | --- | --- | --- | --- | --- | --- | --- | --- |
| Factors | Upgrading | | | | | | Upstaging | | | | | | |
|  | Single regression | | | Multiple regression* | | | Single regression | | | | Multiple regression* | | |
|  | OR | 95% CI | *p* value | OR | 95% CI | *p* value | OR | | 95% CI | *p* value | OR | 95% CI | *p* value |
| Age at surgery |  |  | <0.001 |  |  | <0.001 |  |  |  | 0.004 |  |  | 0.041 |
| continuous | 1.04 | [1.03; 1.06] |  | 1.04 | [1.03; 1.09] |  |  | 1.02 | [1.01; 1.03] |  | 1.014 | [1.01; 1.03] |  |
| Family history of PCa (ref: non) |  |  | 0.224 |  |  |  |  |  |  | 0.791 |  |  |  |
| First degree | 0.79 | [0.58; 1.06] |  |  |  |  |  | 1.08 | [0.87; 1.35] |  |  |  |  |
| Hereditary | 0.83 | [0.53; 1.29] |  |  |  |  |  | 1.00 | [0.71; 1.41] |  |  |  |  |
| Fatal family history of PCa (ref: non) |  |  | 0.090 |  |  |  |  |  |  | 0.696 |  |  |  |
| Yes | 0.54 | [0.26; 1.10] |  |  |  |  |  | 1.09 | [0.71; 1.69] |  |  |  |  |
| Other cancer family history (ref: non) |  |  | 0.603 |  |  |  |  |  |  | 0.587 |  |  |  |
| Yes | 1.06 | [0.85; 1.31] |  |  |  |  |  | 1.05 | [0.88; 1.25] |  |  |  |  |
| Secondary urologic cancer (ref: non) |  |  | 0.639 |  |  |  |  |  |  | 0.881 |  |  |  |
| Urologic cancer | 0.86 | [0.46; 1.61] |  |  |  |  |  | 0.96 | [0.60; 1.56] |  |  |  |  |
| Secondary non-urologic cancer (ref: non) |  |  | 0.377 |  |  |  |  |  |  | 0.950 |  |  |  |
| Non-urologic cancer | 0.84 | [0.57; 1.24] |  |  |  |  |  | 0.99 | [0.74; 1.33] |  |  |  |  |
| PSA at diagnosis (ng/mL) |  |  | <0.001 |  |  | <0.001 |  |  |  | <0.001 |  |  | <0.001 |
| continuous | 1.07 | [1.03; 1.10] |  | 1.06 | [1.03; 1.09] |  |  | 1.11 | [1.08; 1.1] |  | 1.11 | [1.08; 1.13] |  |
| DRE (ref: non-suspicious) |  |  | 0.131 |  |  |  |  |  |  | 0.047 |  |  | 0.030 |
| suspicious | 1.21 | [0.94; 1.56] |  |  |  |  |  | 0.80 | [0.64; 0.99] |  | 0.78 | [0.62; 0.98] |  |
| OR = Odds Ratio; CI = Confidence Interval; PCa = Prostate cancer; PSA = Prostate-specific antigen; DRE = Digital rectal examination  *with backward elimination (selection level 5%) | | | | | | | | | | | | | |
