# Supplementary material for: No detrimental effect of a positive family history on postoperative upgrading and upstaging in men with low risk and favourable intermediate-risk prostate cancer: implications for active surveillance
Source: World J Urol. 2020 Oct 13;39(7):2499–506. doi: 10.1007/s00345-020-03485-5 (PMC8332649; doi:10.1007/s00345-020-03485-5)
Supplement: Supplementary file 2 — Supplementary file2 (DOCX 14 kb) [file 345_2020_3485_MOESM2_ESM.docx]

| **Supplementary Table 2** Single and multiple regression analysis of upgrading from Gleason Grade Group 1 to Gleason Grade Group 2 | | | | | | |
| --- | --- | --- | --- | --- | --- | --- |
| Factors | Upgrading | | | | | |
|  | Single regression | | | Multiple regression* | | |
|  | OR | 95% CI | *p* value | OR | 95% CI | *p* value |
| Age at surgery |  |  | 0.141 |  |  |  |
| continuous | 0.99 | [0.98; 1.01] |  |  |  |  |
| Family history of PCa (ref: non) |  |  | 0.035 |  |  |  |
| First degree | 1.20 | [0.97; 1.50] |  |  |  |  |
| Hereditary | 0.74 | [0.53; 1.04] |  |  |  |  |
| Fatal family history of PCa (ref: non) |  |  | 0.246 |  |  |  |
| Yes | 0.77 | [0.49; 1.20] |  |  |  |  |
| Other cancer family history (ref: non) |  |  | 0.339 |  |  |  |
| Yes | 0.92 | [0.78; 1.09] |  |  |  |  |
| Secondary urologic cancer (ref: non) |  |  | 0.360 |  |  |  |
| Urologic cancer | 0.80 | [0.49; 1.29] |  |  |  |  |
| Secondary non-urologic cancer (ref: non) |  |  | 0.011 |  |  | 0.014 |
| Non-urologic cancer | 0.67 | [0.50; 0.91] |  | 0.68 | [0.51; 0.93] |  |
| PSA at diagnosis (ng/mL) |  |  | <0.001 |  |  | <0.001 |
| continuous | 1.05 | [1.03; 1.07] |  | 1.05 | [1.02; 1.07] |  |
| DRE (ref: non-suspicious) |  |  | 0.146 |  |  |  |
| suspicious | 0.86 | [0.70; 1.05] |  |  |  |  |
| OR = Odds Ratio; CI = Confidence Interval; PCa = Prostate cancer; PSA = Prostate-specific antigen; DRE = Digital rectal examination  *with backward elimination (selection level 5%) | | | | | | |
